# Effect of Temperature on the Sliding Wear Behaviors of Carburized BG801 Bearing Steel

**DOI:** 10.3390/ma19051034

**Published:** 2026-03-08

**Authors:** Qiongdi Wang, Zhaojie Meng, Shuangyan Qi, Chunyang Luo, Xiuhua Guo, Zhaodong Wang, Kexing Song

**Affiliations:** 1 Institute of Materials, Henan Academy of Sciences, Zhengzhou 450046, China; 15236325684@163.com (Q.W.); qsyan2002@163.com (S.Q.); kxsong@haust.edu.cn (K.S.); 2 School of Materials Science and Engineering, Henan University of Science and Technology, Luoyang 471023, China; guoxiuhua@haust.edu.cn; 3 State Key Laboratory of Digital Steel, Northeastern University, Shenyang 110819, China; luochunyang2020@163.com

**Keywords:** bearing steel, vacuum low-pressure carburizing, elevated-temperature friction, wear mechanisms

## Abstract

**Highlights:**

**What are the main findings?**

- Vacuum low-pressure carburizing is adopted to optimize the surface properties of BG801 bearing steel, overcoming the adaptability constraints of conventional carburizing processes and offering a novel technical route for improving its performance.
- Targeting the actual bearing service temperature range of 25~400 °C, this work systematically compares the tribological properties of carburized and uncarburized specimens and reveals the wear-resistant mechanism of the synergistic martensite–carbide layer, deepening the tribological theory of carburized bearing steel.
- Carburizing reduces the wear rate of BG801 by approximately 50% and markedly enhances its high-temperature wear resistance.

**What are the implications of the main findings?**

- The research balances theoretical significance and engineering practicality, providing a feasible solution for the application and industrial promotion of BG801 in medium-to-high-temperature conditions.
- The wear-resistant mechanism of the synergistic martensite–carbide layer provides a theoretical basis for the design of high-temperature wear-resistant bearing steels.
- Vacuum low-pressure carburizing offers a promising approach to enhance the surface performance of other bearing steels under harsh service conditions.

**Abstract:**

The wear performance of BG801 bearing steel under elevated-temperature conditions exerts a decisive influence on the service life and operational reliability of aero-engine bearings. In this study, the vacuum low-pressure carburizing heat treatment technology was employed to perform surface carburization on BG801 bearing steel, and the effect of carburization on the frictional properties of this steel was explored over a temperature range of 25 °C to 400 °C. The results indicate that with the increase in temperature, the friction coefficients of both the uncarburized specimens (hereinafter referred to as BG801-NC) and carburized specimens (hereinafter referred to as BG801-C) are maintained in the range of 0.5~0.8. Compared with BG801-NC, the wear rate of BG801-C is reduced by approximately 50% and exhibits an overall variation tendency of increasing first and then decreasing. At elevated temperatures, BG801-C presents superior wear resistance, which is attributed to the formation of a martensite–carbide composite strengthened layer on the surface of the bearing steel after carburizing treatment, a microstructure that remarkably enhances the surface hardness and wear resistance of the steel. Moreover, the carburized layer also diminishes the thickness of the plastic deformation layer during the friction process, thereby further suppressing the extension of wear damage.

## 1. Introduction

Bearings serve as critical load-bearing components within the aero-engine rotor system. Some of these bearings are located adjacent to the high-temperature core engine region, operating in environments ranging from approximately 170~350 °C [1]. Consequently, their wear and failure behavior under such elevated temperatures directly dictates the safety and service life of the engine. This imposes stringent demands on the performance of bearing steels under extreme thermal conditions. In response to these requirements, the bearing steel BG801 (internationally designated as CSS-42L) was developed specifically to meet such challenges [2]. As one of the typical representatives of third-generation aviation bearing steel, BG801 exhibits superior strength, hardness and contact fatigue strength [3,4,5] and can therefore be widely applied in fields such as high-end machinery manufacturing, precision transmission systems, automotive components, and aerospace equipment [6,7]. In practical applications, BG801 bearing steel is prone to surface wear and fatigue spalling under demanding operating conditions involving elevated temperatures and high-frequency cyclic loading. These limitations constrain its broader adoption in more advanced equipment [8,9].

Numerous studies have demonstrated that surface modification treatment can effectively enhance the surface properties of BG801 bearing steel to meet the performance demands under severe working conditions [10,11,12]. Song et al. [13] constructed a gradient nanostructure on BG801 steel via 15 min ultrasonic shot peening treatment, imparting the steel with significantly superior wear resistance compared with the untreated coarse-grained steel; in the dry sliding wear test at 500 °C, the wear rate was reduced by 80.42% compared with the as-received state. Lin et al. [14] deposited TiAlMoNbW high-entropy alloy (HEA) and high-entropy nitride (HEN) films on the surface of BG801 bearing steel via magnetron sputtering. Results indicated that the introduction of nitrogen could densify the film structure, reduce surface roughness, and enhance the wear resistance of the substrate, while also providing excellent corrosion resistance. Furthermore, Xue et al. [15] demonstrated that BG801 steel, featuring surface texturing filled with Sn-Ag-Cu-Ti_3_C_2_, exhibited outstanding friction-reducing and anti-wear properties under elevated temperatures and severe lubrication conditions. However, despite the significant effectiveness of these modification methods, the resulting modified layer is relatively thin, which fails to satisfy the stringent requirements for high load capacity and long service life demanded of BG801 bearing steel.

Compared with other surface treatment technologies, carburizing treatment can significantly optimize the surface microstructure and stress state of BG801 bearing steel by precisely regulating the carbon concentration distribution in the material’s surface layer [16,17], greatly enhancing the material’s surface hardness, wear resistance and contact fatigue performance [18]. Lin et al. [19] demonstrated that after carburizing treatment, uniformly dispersed and fine M_7_C_3_ carbides are formed in the subsurface of BG801 bearing steel. Meanwhile, the matrix grains are significantly refined, which substantially enhances wear resistance. Wang et al. [20] revealed that carbon ion implantation can effectively enhance the corrosion resistance and wear resistance of BG801 steel. This treatment method does not affect the physical and mechanical stability of the material; thus, it can significantly improve the surface properties without sacrificing the overall performance of the material [21,22,23]. It is worth noting that the aforementioned studies mostly focus on surface treatments such as nanocrystallization or ion implantation. However, research on the mechanism underlying the wear and failure resistance of the carburized layer in BG801 steel remains critical to understanding its high-temperature service performance.

Based on the above considerations, vacuum low-pressure carburizing was adopted for the surface modification of BG801 steel in this study, and friction and wear tests were conducted on both BG801-C and BG801-NC across a temperature range of 25 to 400 °C. Using characterization techniques including scanning electron microscopy (SEM), transmission electron microscopy (TEM), and energy-dispersive X-ray spectroscopy (EDS), the effect of the carburized layer microstructure on the high-temperature wear behavior was elucidated. This work thereby provides experimental support for the application of carburized BG801 bearing steel in elevated-temperature environments.

## 2. Experimental Procedure

### 2.1. Material Preparation

**Figure 1:**
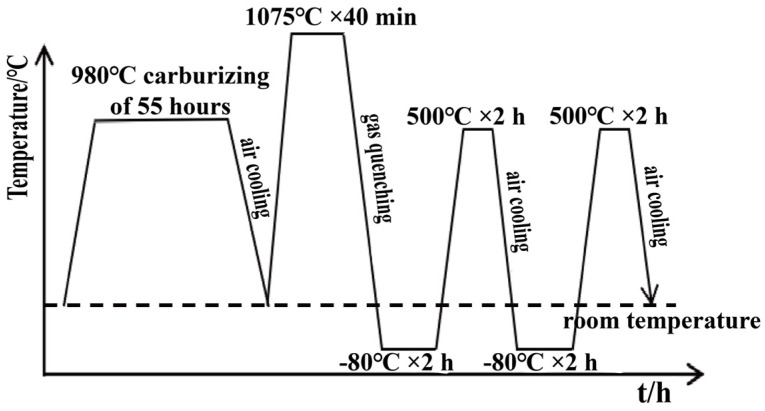
Schematic diagram of the heat treatment process.

In this study, BG801 aero-engine bearing steel was selected as the experimental material, and its chemical composition is listed in Table 1. The specimens were subjected to carburizing treatment using a vacuum low-pressure carburizing apparatus (Dongbo, Model DB-644, Shenyang, China). Based on preliminary experiments, the optimal parameters were selected as shown in Figure 1 [24,25]. The specific process parameters are as follows: Carburizing was conducted at 980 °C for 55 h, followed by gas quenching. Subsequently, secondary quenching was performed at 1075 °C for 40 min, also followed by gas quenching. Finally, tempering was carried out at 500 °C for 2 h in a box-type resistance furnace with subsequent air cooling, and cryogenic treatment was performed in a chamber that was continuously supplied with liquid nitrogen.

### 2.2. Surface Morphology Analysis

After heat treatment, the surface characteristics of BG801-NC and BG801-C were investigated. All specimens were mechanically ground using sandpaper ranging from 120# to 2000# and then treated separately according to different test requirements. For microstructure observation, the specimens were mechanically polished with 2.5 μm diamond polishing paste and subsequently etched with a solution consisting of 1 g CuCl_2_, 3.5 g FeCl_3_, 50 mL HCl, 2.5 mL HNO_3_, 50 mL ethanol and 50 mL distilled water [26]. For X-ray diffraction (XRD) tests, the electrolyte was composed of perchloric acid and methanol with a volume ratio of 1:9, and the tests were conducted at 20 V and −30 °C for 30 s. Subsequently, XRD measurements (Malvern Panalytical, Empyrean, Almelo, Netherlands) were performed with a scanning range (2θ) of 35° to 90° and a scan speed of 2°/min [27,28]. Additionally, Electron Backscatter Diffraction (EBSD) analysis was carried out using an in situ scanning electron microscope (Qiyue Technology, In-situ SEM 340FC, Zhejiang, China). Hardness was measured using a fully automatic Vickers hardness tester (EVERYONE, VH500-3A, Shanghai, China). Finally, surface roughness was evaluated via Atomic Force Microscopy (Bruker, Dimension Icon, Billerica, MA, USA), thereby achieving a comprehensive multi-dimensional characterization of the specimens.

### 2.3. Friction Tests

**Figure 2:**
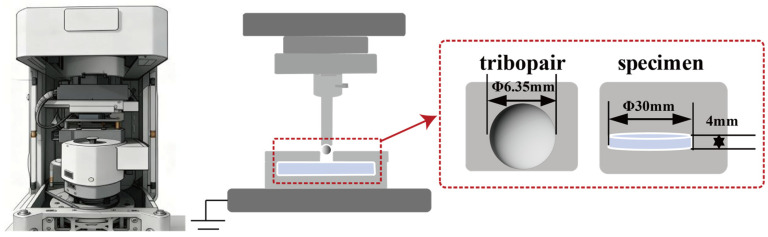
Multifunctional friction and wear tester and schematic of specimen dimensions.

The elevated-temperature friction and wear tests were conducted using a multifunctional friction tester (Bruker, Model UMT-2, Campbell, CA, USA) in the ball-on-disk configuration (Figure 2). The tribopair was selected as a ZrO_2_ ceramic ball with a diameter of 6.35 mm (hardness > 1200 HV); the substrate was a BG801 bearing steel disk with dimensions of ϕ30 mm × 4 mm. Prior to testing, the specimens were ground using 2000# sandpaper and ultrasonically cleaned in anhydrous ethanol for 15 min and dried. The experimental parameters were set as follows: 10 N of friction load, 200 r/min of sliding speed, 4 mm of rotational radius, 60 min of test duration, and test temperatures of 25 °C, 100 °C, 200 °C, 300 °C, and 400 °C, respectively.

### 2.4. Wear Surface Tests

After the friction tests, the wear tracks were scanned using a white-light interferometer (Bruker, Model ContourX-200, Tucson, AZ, USA) to obtain their 3D topographies and wear volumes. The wear rate (W) was calculated using Equation (1) [29,30].

The calculation formula is given as follows:

ω = ∆υ/2πrnF(1)

where ω (mm^3^/(N·m)) is the wear rate; ΔV (mm^3^) is the wear volume; n is the total number of revolutions; r (mm) is the rotational radius; and F (N) is the applied load.

Furthermore, the morphologies and elemental distributions of the worn surfaces were characterized using a scanning electron microscope (ZEISS, EVO10, Oberkochen, Germany). Additionally, the subsurface microstructures of the wear tracks formed at 25 °C and 400 °C were analyzed using a transmission electron microscope (FEI, Model Talos F200X G2, Hillsboro, OR, USA).

## 3. Results

### 3.1. Surface Analysis

#### 3.1.1. Microstructural Morphological Characteristics

**Figure 3:**
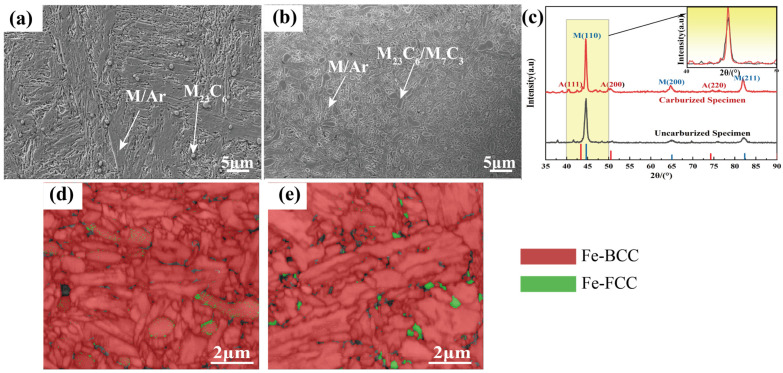
Microstructures, XRD patterns, and EBSD maps of BG801 bearing steel: (**a**,**d**) BG801-NC; (**b**,**e**) BG801-C; (**c**) Comparison of XRD patterns between BG801-C and BG801-NC.

Figure 3 presents the microstructure, phase composition and crystallographic characteristics of BG801-NC and BG801-C. The SEM morphology of BG801-NC (Figure 3a) shows a microstructure primarily consisting of uniform lath martensite, a small amount of retained austenite, and dispersed carbides [31]. The corresponding EBSD phase distribution map (Figure 3d), where martensite (Fe-BCC) and austenite (Fe-FCC) are denoted by red and green, respectively, confirms that martensite is the dominant phase, while austenite exists as dispersed fine particles. After carburizing treatment, the surface microstructure of BG801 bearing steel (Figure 3b) undergoes a significant transformation, meaning that a high density of fine granular cementite precipitates is revealed in SEM images. In addition, the martensite is notably refined under the induction of high carbon concentrations, and the content of retained austenite increases significantly. The EBSD phase distribution map (Figure 3e) further quantifies this evolutionary process, showing a substantial increase in the volume fraction of surface-retained austenite, which is interleaved with refined martensite and cementite. XRD results (Figure 3c) corroborated these observations. The diffraction pattern of BG801-NC is dominated by martensite peaks with minor cementite peaks, whereas BG801-C exhibits enhanced austenite diffraction peaks and a marked increase in the intensity of cementite diffraction peaks.

#### 3.1.2. Microhardness Analysis

**Figure 4:**
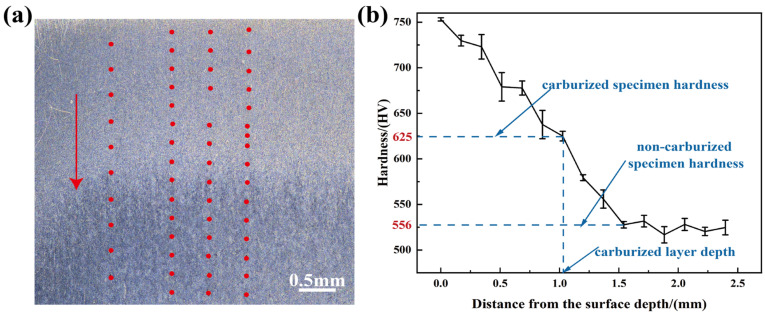
Hardness gradient and cross-section of BG801 bearing steel: (**a**) cross-section image (from the carburized surface toward the core); (**b**) hardness profile.

Microhardness profiles were characterized along the cross-sections of BG801 bearing steel specimens, as shown in Figure 4. BG801-NC exhibited a surface hardness of approximately 556 HV. After carburization, the surface hardness increased to about 625 HV, with a carburized layer depth of 1.2 mm. These results confirm that carburization can significantly improve the surface hardness of BG801 bearing steel compared with BG801-NC.

According to the results presented in Table 1, cobalt (Co), molybdenum (Mo), and chromium (Cr) contribute to hardness enhancement and regulated carbide formation through solid-solution strengthening and dispersion strengthening [32]. The high hardness of BG801-C essentially arises from the dual strengthening of the high-carbon martensitic matrix and finely dispersed carbides. Although the retained austenite content in BG801-C is slightly higher than that in BG801-NC, its overall fraction is limited, thermally stable, and homogeneously distributed. Furthermore, the retained austenite can undergo local transformation hardening via the TRIP (Transformation-Induced Plasticity) effect during deformation, which not only maintains the surface hardness but also further improves the toughness and fatigue life of the carburized layer.

#### 3.1.3. Surface Roughness Analysis

**Figure 5:**
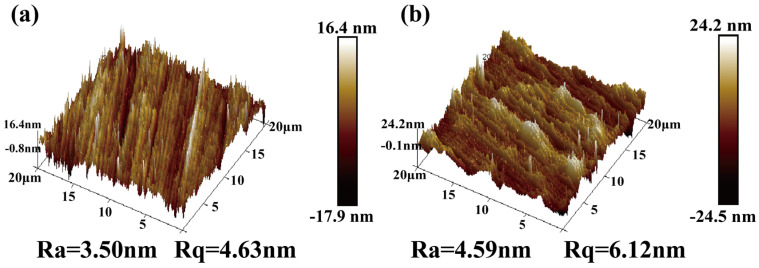
3D surface topography maps of BG801 bearing steel: (**a**) BG801-NC; (**b**) BG801-C.

Figure 5 presents the surface roughness analysis of BG801-NC and BG801-C. As can be seen from the figure, BG801-NC (Figure 5a) exhibits a compact microstructure without pores and obvious distortion induced by phase transformation, and its surface morphology is relatively flat, with an Ra value of 3.5 nm. After carburizing treatment (Figure 5b), the reconstruction of martensite and cementite in the surface layer induces grain boundary distortion and volume expansion, which results in an increase in surface micro-protrusions and peak-to-valley differences; the Ra value rises to 4.59 nm, indicating that the surface roughness of BG801-C is significantly higher than that of the uncarburized one.

### 3.2. Friction and Wear Properties Analysis

#### 3.2.1. Friction Coefficient

**Figure 6:**
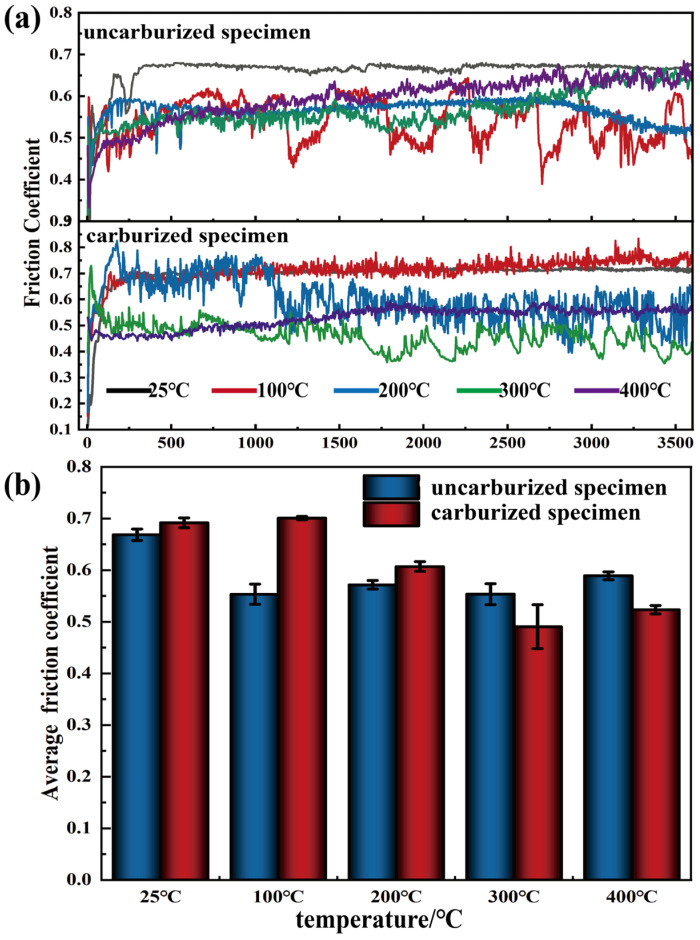
Friction coefficients of BG801 bearing steel: (**a**) typical friction coefficient curves; (**b**) average friction coefficient.

Tribological tests were carried out on both BG801-C and BG801-NC specimens at 25, 100, 200, 300, and 400 °C, with at least three parallel tests at each temperature. Typical friction coefficient curves for each group are plotted in Figure 6a. The average friction coefficients and standard deviations (SDs) were calculated from the steady-state stage to characterize data dispersion, and the results with error bars are presented in Figure 6b.

As illustrated in Figure 6a, the typical friction coefficient of BG801 bearing steel initially increases, then decreases slightly, and finally stabilizes with increasing sliding time. The initial stage corresponds to the running-in period, corresponding to the mutual adaptation process between the ZrO_2_ tribopair and the BG801 bearing steel. During this stage, the friction coefficient shows large fluctuations. However, as sliding continues, the protrusions on the contact surface gradually decrease or even disappear, the friction coefficient gradually stabilizes, and the friction system eventually enters the steady-state stage. Notably, the friction coefficient curves at elevated temperatures (e.g., 300 °C and 400 °C) exhibit shorter running-in periods and smaller fluctuations. This phenomenon can be attributed to the thermal softening effect of the specimen surface, which shortens or even eliminates the running-in adaptation period of the friction process.

Figure 6b presents the average friction coefficients of the two types of specimens at different test temperatures. With the increase in test temperature, the average friction coefficient of BG801-NC initially decreases and then increases. Conversely, BG801-C exhibits the opposite trend, initially increasing, followed by a decrease. Specifically, BG801-NC with lower hardness exhibits a faster surface evolution rate; a thin lubricating layer can be formed at low temperatures, leading to the lowest friction coefficient, while with increasing temperatures, the surface layer thickens and its stability decreases, and the increased interfacial shear resistance results in a higher friction coefficient. In contrast, BG801-C with high hardness and carbide strengthening possesses superior contact load-bearing capacity and more favorable high-temperature contact mechanics; owing to its slower surface evolution rate, it is difficult to form an effective lubricating layer at low temperatures, leading to a relatively high friction coefficient, but above 200 °C, a compact, thermally stable and alloy-enriched third-body glaze layer is formed, which provides excellent lubrication and causes a significant reduction in the friction coefficient.

#### 3.2.2. Wear Rate

**Figure 7:**
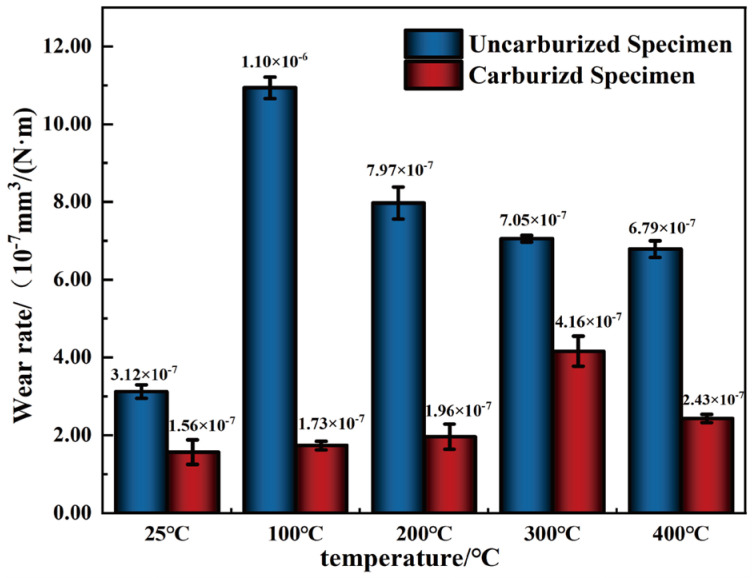
Wear properties of BG801 bearing steel.

Figure 7 illustrates the evolution of the wear rate of BG801 bearing steel with temperature. Clearly, the wear behavior of BG801 bearing steel is significantly influenced by both temperature and carburizing treatment. For BG801-NC, the wear rate first increases and then decreases, presenting an abnormal behavior at 100 °C, where the friction coefficient is the lowest. This is because the oxide film formed at this temperature is thin, brittle and weakly bonded to the substrate, as well as prone to cracking and delamination during friction, and thus cannot effectively protect the substrate, resulting in severe material loss. With increasing temperatures, the protective effect of the oxide film is gradually strengthened, and the wear rate consequently decreases. In contrast, the wear rate of BG801-C exhibits a three-stage trend: an initially low value, followed by a slight increase, and a subsequent decline. The wear rate is only approximately 1.56 × 10^−7^ mm^3^/(N·m) at 25 °C, indicating that the high hardness of the carburized layer effectively inhibits wear damage. With the increase in test temperature, the wear rate increases slightly, which is mainly attributed to minor thermal softening of the specimen surface, which results in increased wear loss. Notably, the wear rate of BG801-C remains significantly lower than that of BG801-NC over the entire temperature range. This is mainly ascribed to the high hardness induced by carbide strengthening in the carburized layer.

#### 3.2.3. Friction Morphology Analysis

(1) Three-dimensional Topographies of The Wear tracks

**Figure 8:**
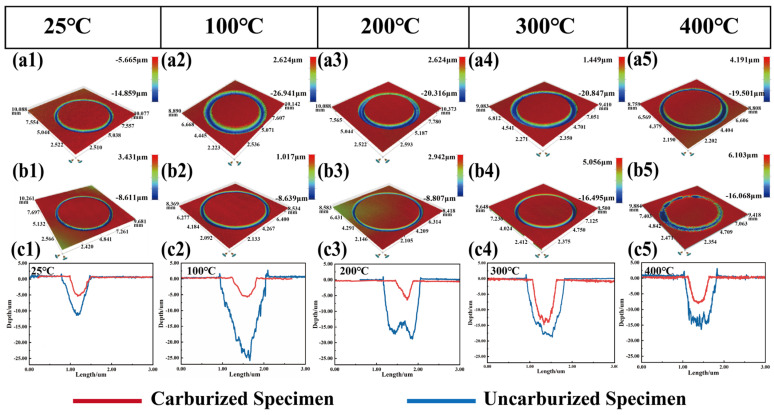
3D topographies and wear track depths of BG801 bearing steel: (**a1**–**a5**) BG801-NC; (**b1**–**b5**) BG801-C; (**c1**–**c5**) statistical analysis of wear track depths.

To accurately characterize the wear track morphology, a non-contact white-light interferometer was employed to analyze the wear tracks, and the relevant results are presented in Figure 8(a1–a5,b1–b5). The color gradient in the wear track corresponds to variations in wear depth. Irregular regions are observed in the center of the wear tracks. Additionally, the edges of the wear track are slightly raised above the specimen surface, which is primarily caused by the accumulation of wear debris at the wear track edges.

Figure 8(c1–c5) illustrates the cross-sectional profiles and wear depths of the specimens at different temperatures. For BG801-NC, the depth and width of the wear tracks initially increase and then decrease with the increasing test temperature. The maximum wear depth of both specimens appears at a test temperature of 100 °C. In contrast, the depth and width of the wear tracks on BG801-C increase gradually with rising test temperatures, with a maximum depth of 17.2 μm. At any given test temperature, both the depth and width of the wear tracks on BG801-C are significantly smaller than those of BG801-NC, which indicates that BG801-C possess superior wear resistance.

(2) Wear Track Morphology

**Figure 9:**
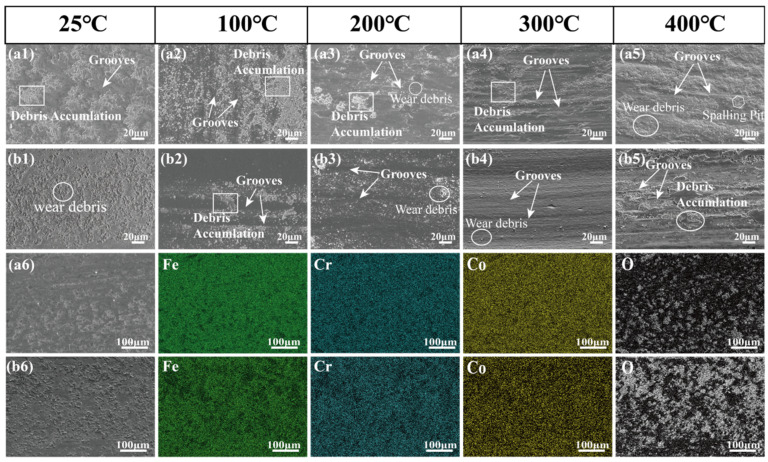
SEM images and EDS analyses of worn surfaces at different test temperatures: (**a1**–**a6**) BG801-NC; (**b1**–**b6**) BG801-C.

Figure 9 presents the morphologies and EDS results for wear tracks on the surface of BG801-NC and BG801-C after friction tests at different temperatures. Figure 9(a1–a6) display SEM micrographs of the wear tracks on BG801-NC; in the low-temperature range (25~100 °C), the worn surface exhibits obvious blocky spalling pits, severe plowing grooves, and associated tearing marks. Integrated with the corresponding EDS analysis (Table 2), it can be found that the oxygen (O) content decreased from 10.53% at 25 °C to 7.56% at 100 °C, which indicates that the newly formed oxide layers were rapidly removed by severe mechanical wear. In contrast, the iron (Fe) content increases from 54.6% to 58.05%, which corresponds to the significant accumulation of metallic debris induced by adhesive wear. Therefore, the dominant wear forms of BG801-NC in the 25~100 °C range are mixed with adhesive wear and abrasive wear. In the medium-to-high temperature range (200~400 °C), loose flocculent oxide debris accumulates in the wear tracks, and apparent wavy plowing grooves were observed at 400 °C. Correspondingly, the O content increased continuously from 10.75% to 19.55%, indicating that the wear mechanism was gradually dominated by oxidation wear.

Figure 9(b1–b6) show SEM images of the wear tracks on BG801-C. At a test temperature of 25 °C, the worn surface exhibits numerous shallow and fine plowing grooves and oxide debris, indicating that the wear mechanism is dominated by mild abrasive wear. In the test temperature range of 100~300 °C, only a small amount of oxide debris is present on the wear track, and the worn surface remains smooth. Thus, the wear mechanism is still dominated by abrasive wear. However, when the test temperature reaches 400 °C, surface oxidation is intensified, and the spalling pits appear. Consequently, the wear mechanism transitions to a combination of oxidative wear and abrasive wear. This indicates that the wear mechanism of BG801-C is dominated by mild abrasive wear accompanied by oxidation wear over the entire test temperature range.

In summary, under test conditions from 25 °C to 400 °C, the wear mechanism of BG801-NC transitions from a mixture of adhesive and abrasive wear to oxidation-dominated wear with increasing temperatures. In contrast, the carburizing treatment significantly enhances the surface strength, enabling BG801 bearing steel to maintain excellent wear resistance at operating temperatures ranging from 100 °C to 300 °C. Furthermore, it increases the critical temperature of severe wear damage from 200 °C to 400 °C, thereby significantly enhancing the wear resistance under elevated-temperature service conditions.

(3) Worn Surface Characteristics of tribopairs

**Figure 10:**
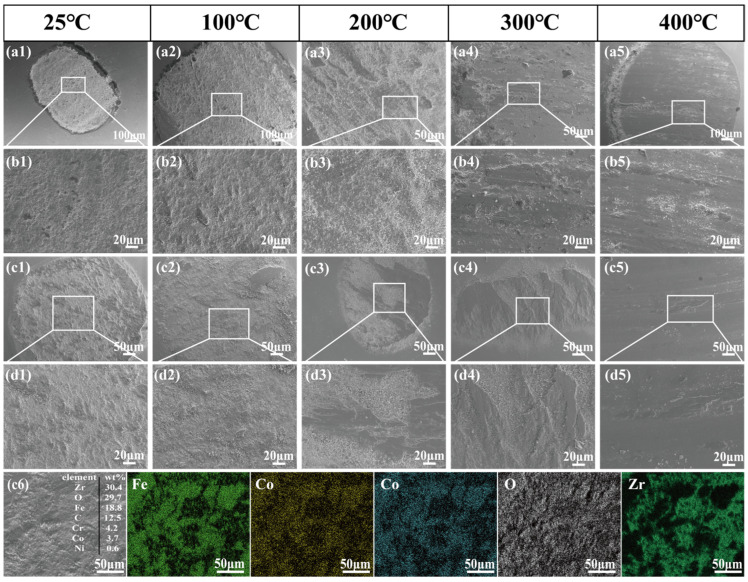
SEM images of ZrO_2_ tribopairs at different test temperatures and EDS analyses of the ZrO_2_ tribopairs combined with BG801-NC at 25 °C: (**a1**–**b5**) ZrO_2_ vs. BG801-NC; (**c1**–**d5**): ZrO_2_ vs. BG801-C.

To further investigate the effect of temperature on material transfer behavior, the worn surfaces of the ZrO_2_ tribopairs were characterized by SEM and EDS at different test temperatures, and the corresponding results are presented in Figure 10. During friction, owing to the significantly higher hardness of ZrO_2_ than that of BG801 bearing steel, wear debris from the bearing steel continuously transfers onto ZrO_2_ and forms a complex adhesive layer through interfacial adsorption. EDS analysis (Figure 10(c6)) reveals that the adhered materials mainly consist of Fe, C, and Cr (which is similar to the nominal composition of BG801), with a high oxygen content, indicating that oxidation occurs during sliding. Given the high chemical stability of ZrO_2_, elevated temperatures accelerate the oxidation of the bearing steel. The transferred wear debris is oxidized and compacted into an oxide/glaze layer, which consumes the loose metallic adhesive layer and decreases its content on the tribopairs with increasing temperatures [33]. Specifically, a distinct adhesive layer is observed on the ZrO_2_ tribopairs paired with BG801-NC (Figure 10(a1–b5)), whose morphology is similar to delaminated wear debris in the wear track under high magnification. In comparison, blocky adhesions exist on the ZrO_2_ tribopair paired with BG801-C (Figure 10(c1–d5)), but with a smaller contact area. This is attributed to the higher surface hardness (625 HV) and uniformly dispersed carbides in BG801-C, which generate finer and more homogeneous wear debris during friction and facilitates the formation of a dense and continuous glaze layer at high temperatures, with strong interfacial bonding between the oxide layer and the substrate. Under the same load, the contact region experiences slight plastic deformation, corresponding to a smaller indentation diameter on the tribopairs. In contrast, BG801-NC with lower hardness is prone to significant plastic flow, thereby generating coarse, loose wear debris that results in a porous layered structure, resulting in a larger indentation diameter on the tribopairs.

#### 3.2.4. Structural Evolution of Wear Scar’s Subsurface

**Figure 11:**
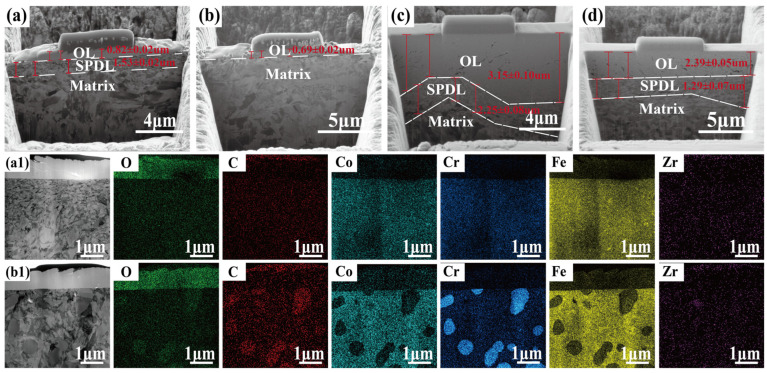
TEM images of wear track subsurface and SEM images and EDS analyses of friction-affected layers at 25 °C: (**a**,**a1**) BG801-NC at 25 °C; (**b**,**b1**) BG801-C at 25 °C; (**c**) BG801-NC at 400 °C; (**d**) BG801-C at 400 °C.

Transmission electron microscopy (TEM) analysis was employed to examine cross-sectional specimens extracted from the wear tracks to elucidate the microstructural evolution induced by sliding friction, particularly the formation of subsurface deformation layers. As shown in Figure 11, the friction-affected zone can be categorized into three sublayers based on microstructural characteristics, extending from the surface layer to the internal substrate: the oxidation layer (OL), the severe plastic deformation layer (SPDL), and the matrix [15]. The morphology and thickness of these friction layers are observed to undergo significant changes with test temperature and specimen type. When the temperature is kept constant, BG801-NC exhibits greater thicknesses of both the OL and SPDL compared to BG801-C. For BG801 bearing steel, a consistent trend is observed, in which the thicknesses of the OL and SPDL increase with increasing test temperatures, regardless of carburization state.

Under the dry sliding friction condition at 25 °C, BG801-NC (Figure 11(a,a1)) exhibits a non-uniform oxidation layer, a deeply penetrating severe plastic deformation layer, and distinct distortion of the underlying substrate grains, which is induced by the deformation penetration effect. In contrast, BG801-C (Figure 11(b,b1)) possesses a uniform and dense oxidation layer, accompanied by markedly attenuated grain deformation, by virtue of the surface Cr-based carbides that impede dislocation slip and grain boundary migration [34,35].

When the friction temperature is elevated to 400 °C, the oxide layer on BG801-NC (Figure 11c) develops through-thickness cracks induced by thermal expansion mismatch and cyclic mechanical impact. These cracks subsequently extend into the underlying SPDL. In contrast, BG801-C (Figure 11d) features a surface layer with high hardness and carbide reinforcement beneath the dense interfacial glaze/oxide layer, and these two components collectively form a “double-buffering” mechanism. Specifically, the high-hardness carbide layer refines and homogenizes the SPDL, thereby effectively dispersing stress, cushioning impact, and suppressing microcrack initiation. Meanwhile, the dense interfacial glaze layer allows for a smooth transition of interfacial shear stress and mitigates local damage. The synergistic effect of these two layers endows the material with excellent high-temperature tribological properties.

**Figure 12:**
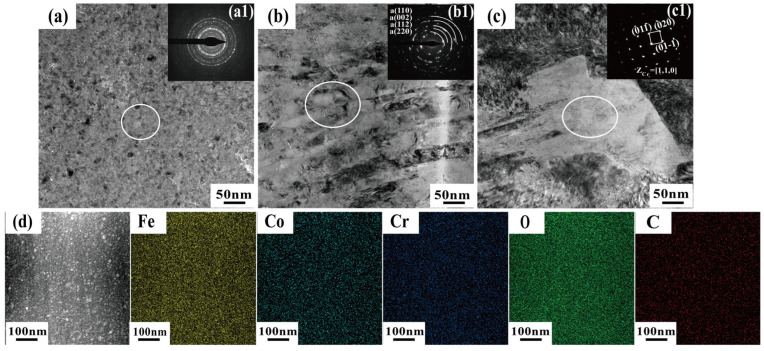
TEM images of wear tracks’ subsurfaces and EDS analysis of the oxidation layer of BG801-C at 400 °C: (**a**,**d**) oxidation layer; (**b**) severe plastic deformation layer; (**c**) matrix; (**a1**–**c1**) HRTEM images of the corresponding regions in (**a**–**c**), respectively.

Figure 12 presents the cross-sectional TEM analysis of the wear track on BG801-C after being tested under dry sliding at 400 °C for 60 min. The surface oxide layer (Figure 12a,d) is composed of equiaxed nanoparticles of Co- and Fe-based composite oxides with a grain size of approximately 6 nm. The Cr element from the carburized layer improves the strength and toughness of the oxide layer; however, micropores within the layer can initiate local spallation. Directly beneath the oxide layer lies a nanocrystalline elongated martensitic structure (Figure 12b), which forms a hard transition region that effectively restricts plastic deformation from propagating into the deeper substrate. The Cr-rich [110] single-crystal particles (Figure 12c) that are precipitated within the substrate strengthen the interface and alleviate stress concentration through second-phase strengthening. Ultimately, the tri-layer synergistic structure endows BG801-C with excellent wear resistance under elevated-temperature friction conditions.

## 4. Conclusions

Based on the ball-on-disk sliding wear test, this study systematically investigated the tribological behaviors of BG801-C and BG801-NC under different temperatures, and the principal conclusions are summarized as follows:

1. Vacuum low-pressure carburizing effectively optimizes the surface properties of BG801 bearing steel by forming a martensite–carbide composite layer, which significantly increases surface hardness and thus greatly improves its wear resistance.
2. Compared with BG801-NC, BG801-C achieves a significant improvement in wear resistance, with the wear rate being reduced by approximately 50%. The wear rate of BG801-C shows an overall variation trend of increasing first and then decreasing with the rise in temperature, and it maintains superior wear resistance under elevated-temperature conditions.
3. The dominant wear mechanisms for BG801-NC are severe abrasive wear and adhesive wear, with oxidative wear becoming progressively more pronounced as the temperature rises. In contrast, BG801-C is characterized by a wear mechanism dominated by mild abrasive wear assisted by oxidative wear, achieving a marked reduction in wear severity.
4. The carburized layer of BG801-C can effectively reduce the thickness of the plastic deformation layer that is generated during friction, suppress the formation and extension of wear damage, and further improve the wear resistance and service stability of the steel.

This study overcomes the limitations of conventional carburizing processes for BG801 bearing steel, provides a new technical route for its surface strengthening, and deepens the tribological theory of carburized bearing steels at medium-to-high temperatures. The findings offer important guidance for improving the service life and reliability of aero-engine bearings and lay a solid foundation for the engineering application of BG801 steel under high-temperature conditions.

## Figures and Tables

**Table 1:** Chemical composition of BG801 bearing steel (wt.%).

Material	Cr	Co	Mo	Ni	V	W	C	Fe
BG801	13.97	13.31	4.92	1.91	0.59	0.35	0.14	Bal.

**Table 2:** Elemental contents on wear track surfaces of BG801 bearing steel (wt. %).

Material	wt%	25 °C	100 °C	200 °C	300 °C	400 °C
BG801-NC	O	10.53	7.56	10.75	18.52	19.55
	Fe	54.6	58.05	55.16	50.16	45.86
	Cr	12.22	13.07	12.3	11.33	10.44
BG801-C	O	13.40	5.65	8.40	10.20	12.56
	Fe	52.58	59.55	59.31	59.39	51.72
	Cr	11.57	13.90	14.40	13.69	13.04

## Data Availability

The original contributions presented in this study are included in the article. Further inquiries can be directed to the authors.

## Abbreviations

The following abbreviations are used in this manuscript:

BG801-NC	uncarburized specimen
BG801-C	carburized specimen
SEM	scanning electron microscopy
EDS	energy-dispersive X-ray spectroscopy
TEM	transmission electron microscopy
